# Qualitative Assessment of Network Meta-Analyses of Exercise Therapy for Parkinson's Disease: A Scoping Review

**DOI:** 10.7759/cureus.100412

**Published:** 2025-12-30

**Authors:** Shintaro Ura, Keisuke Ota, Taiyo Kai, Kyohei Sugano, Masaru Narita, Takashi Kitagawa

**Affiliations:** 1 Department of Rehabilitation, Kitano Hospital, Tazuke-Kofukai Medical Research Institute, Osaka, JPN; 2 Department of Rehabilitation, Hokkaido Neurological Hospital, Sapporo, JPN; 3 Department of Rehabilitation Medicine, Nishiyamato Rehabilitation Hospital, Nara, JPN; 4 Department of Rehabilitation, Hyogo Medical University Hospital, Nishinomiya, JPN; 5 Department of Physical Therapy, Shinshu University, Matsumoto, JPN

**Keywords:** exercise therapy/methods, network meta-analysis, parkinson's disease, qualitative assessment, systematic scoping review

## Abstract

The number of network meta-analyses (NMAs) regarding exercise therapy for Parkinson’s disease (PD) has increased rapidly. However, using their findings to inform clinical practice requires confidence in their methodological rigor. This scoping review systematically evaluated the methodological quality and reporting standards of recent NMAs in this field. Comprehensive research was conducted across PubMed, CENTRAL, CINAHL, Web of Science, and other databases through August 31, 2025. Eligible NMAs comparing exercise interventions for PD were assessed using AMSTAR 2 for methodological rigor and PRISMA-NMA for reporting completeness. Twenty-two NMAs were included. While the majority originated from China (68.2%), a sensitivity analysis revealed no systematic methodological differences between Chinese and non-Chinese reviews. Overall methodological confidence was critically low; no NMA achieved a “High” AMSTAR 2 rating, and only three (13.6%) were rated as “Moderate.” Fundamental deficits were prevalent in comprehensive literature searching (Item 4) and the justification for excluded studies (Item 7), with adherence rates of only 9.1% and 4.5%, respectively. Financial transparency was also negligible (<10%). Conversely, adherence to PRISMA-NMA reporting standards was generally satisfactory (>95% for network-specific items), highlighting a discrepancy between reporting compliance and actual study conduct. Current NMAs on exercise for PD exhibit a significant gap between their clinical recommendations and the robustness of the supporting evidence. The widespread lack of comprehensive searching and financial transparency undermines the reliability of these reviews. Future research must prioritize strict adherence to methodological standards over simply completing reporting checklists to provide trustworthy evidence for clinical decision-making.

## Introduction and background

Parkinson’s disease (PD) is the second most common neurodegenerative disorder among adults aged 60 and older [1]. Its cardinal symptoms include motor impairments such as bradykinesia (slowness of initiation and execution of movement), rigidity (muscle stiffness), resting tremor, and postural instability [2]. In addition, non-motor symptoms such as gastrointestinal dysfunction, urinary incontinence, excessive sweating, drooling, and neuropsychiatric disorders may also occur [3]. Motor symptoms often lead to fear of falling, reduced motivation, and inactivity, which may result in social isolation and an increased risk of osteoporosis and cardiovascular disease [4]. Notably, the prevalence of PD is rising faster than that of other neurological diseases [5], with the global population affected projected to double from 6.2 million in 2015 to 12.9 million by 2040 [6], posing a substantial challenge to healthcare systems worldwide.

Exercise therapy is widely used as an adjunctive treatment for PD due to its low cost and accessibility. An expanding body of evidence supports its effectiveness in alleviating both motor and non-motor symptoms, such as slow movement, muscle weakness, and reduced quality of life [7-9]. Recent research has emphasized non-pharmacological and non-surgical interventions, including physiotherapy and other forms of physical exercise. Common modalities range from aerobic, resistance, and mind-body exercises (e.g., Tai Chi) to technology-assisted rehabilitation, targeting key outcomes such as motor function, balance, and daily living activities. A recent systematic review using network meta-analysis (NMA) provided evidence supporting the positive effects of physical exercise in individuals with PD [10].

NMA enables the comparison of multiple interventions by integrating direct and indirect evidence within a single analysis [11]. Simply put, this method relies on the assumption of transitivity (similarity across trials to allow comparison) and the evaluation of inconsistency (discrepancies between direct and indirect evidence) to rank treatments' validity [11]. This methodology allows for estimating comparative efficacy and offers insights that are not achievable through traditional pairwise meta-analysis (MA) [12]. Given its methodological advantages, NMA is considered among the highest levels of evidence [13] and provides valuable information on the effectiveness of exercise interventions for PD.

However, the reporting and analytical quality of NMAs often remain suboptimal [14]. Given the need to broadly map the methodological landscape rather than synthesize efficacy data, a scoping review (ScR) was selected as the most appropriate approach. This review had two primary aims: (i) to conduct a comprehensive qualitative assessment of NMAs focused on exercise therapy for PD, specifically utilizing AMSTAR 2 to evaluate methodological rigor and PRISMA-NMA to assess reporting completeness [15,16], and (ii) to identify areas for improvement based on the findings of this assessment.

## Review

Methods

Study Protocol

This ScR followed the Joanna Briggs Institute Manual for Evidence Synthesis [17] and adhered to the Preferred Reporting Items for Systematic Reviews and Meta-Analyses (PRISMA)-ScR guidelines [18] to ensure methodological rigor (appendices). The study protocol was pre-registered on the Open Science Framework (registration number: https://osf.io/8heka/) [19].

Eligibility criteria included studies involving individuals aged 18 or older with PD. Only NMAs based on randomized controlled trials (RCTs) were included; observational studies were excluded. For transparency, when an NMA's stated eligibility criteria allowed multiple study designs (e.g., RCTs and non-RCTs), we recorded both the stated eligibility and the actual included study designs. However, the determination of eligibility for our scoping review was based on the actual study designs included in the analysis. Eligible NMAs had to compare at least four interventions, including non-exercise control groups or various exercise modalities, with the number of studies exceeding the number of interventions. No restrictions were imposed on language or geographic location.

Exclusion criteria were studies involving atypical Parkinsonism, other neuromuscular disorders, drug or surgical interventions, diagnostic test reliability studies, animal experiments, or NMAs based solely on non-RCTs.

Search Strategy

Eligible study designs included NMAs, mixed-treatment comparison MAs, and multi-treatment MAs. Grey literature, including dissertations and relevant databases, was also considered. Conference abstracts and studies not based on NMA methods were excluded.

Searches were conducted in PubMed, CENTRAL, CINAHL, Web of Science, ClinicalTrials.gov, PEDro, and Epistemonikos, using tailored keyword and MeSH combinations. The literature search was current as of August 31, 2025. Full search strings for all databases are provided in the Appendix to ensure reproducibility. Authors of included studies were contacted when necessary to obtain missing information.

Selection of Sources of Evidence

After deduplication using Rayyan [20], two independent reviewers (S.U. and T.Ka.) screened titles and abstracts. Full texts of potentially eligible articles were then assessed independently by the same reviewers. Disagreements were resolved through discussion or by consulting a third reviewer (T.Ki.). The PRISMA 2020 flow diagram summarizes the selection process [21].

Data Charting Process

Data extraction and quality assessment were conducted independently by four reviewers (S.U., T.K., K.S., and M.N.) using a standardized, pilot-tested data extraction form. Extracted information included author, year, country of the first author, protocol registration, use of PRISMA-NMA guidelines [15], number of included studies, and number of participants. For the quality assessment, reviewers strictly adhered to the official operational guidance documents for AMSTAR 2 and PRISMA-NMA to ensure consistent coding. Discrepancies were resolved by discussion or by consulting a third reviewer.

Critical Appraisal

The AMSTAR-2 tool was used independently by at least two reviewers (S.U., T.K., K.S., M.N., and K.O.) to assess the methodological quality of each included NMA [16]. Overall confidence was categorized as high, moderate, low, or very low based on the assessment of critical domains. PRISMA-NMA adherence was evaluated individually for NMAs that did not submit a completed checklist. Shea et al. stated in their methods that both randomized and non-randomized controlled trials would be eligible, but in practice, only randomized controlled trials were included. Because the rationale for including multiple study designs was not explicitly explained, we rated AMSTAR 2 item 3 as “No.”

Data Synthesis and Analysis

We synthesized the data descriptively. AMSTAR 2 ratings are reported as counts and percentages (n/N), whereas PRISMA-NMA compliance is presented as adherence rates (%). We also conducted descriptive subgroup comparisons to examine differences based on geographic origin (China vs. other regions) and checklist submission status; statistical hypothesis testing was not applied.

Results

Literature Search

**Figure 1:**
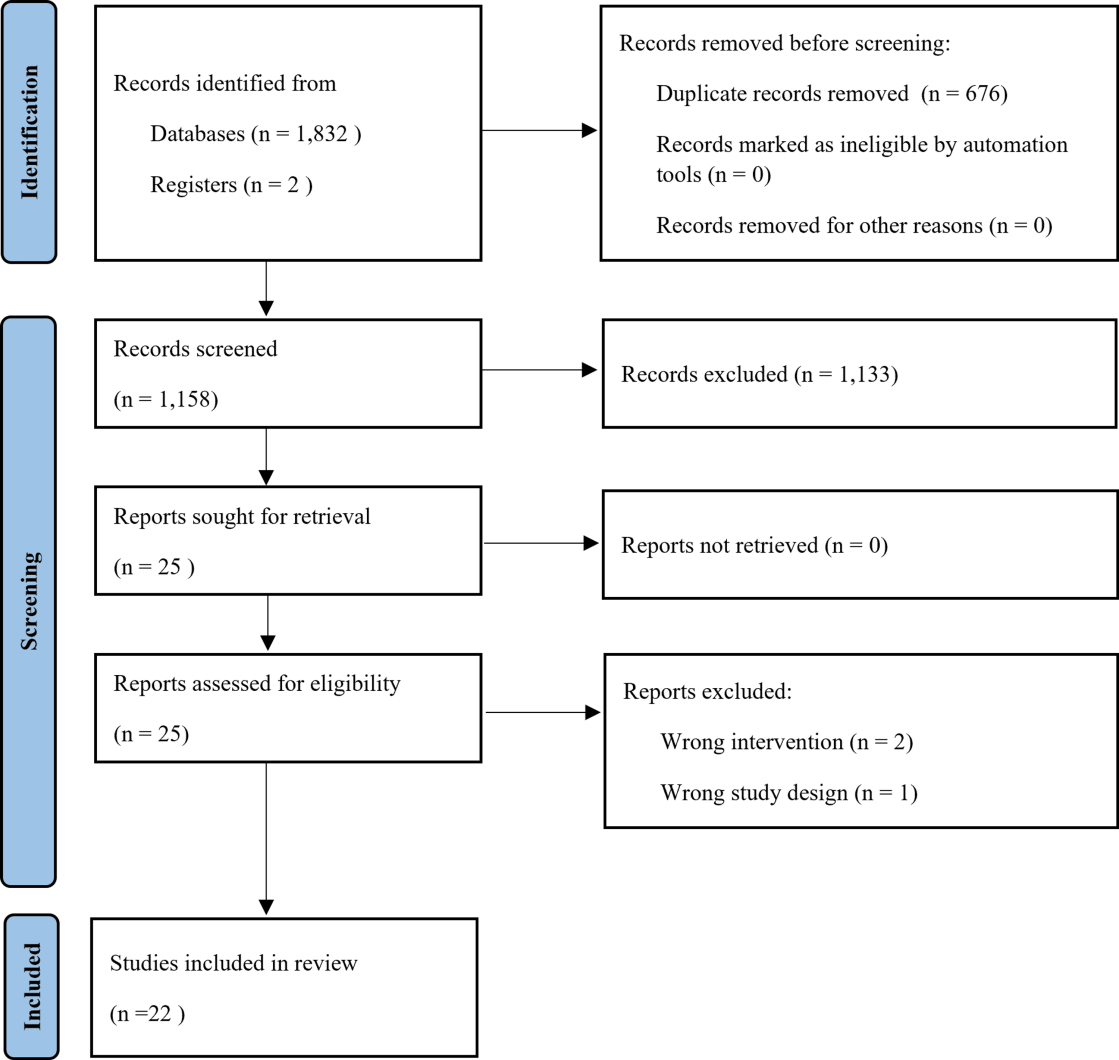
PRISMA flowchart PRISMA: Preferred Reporting Items for Systematic Reviews and Meta-Analyses

The search yielded 1,834 citations, with 1,158 titles and abstracts screened after deduplication (Figure 1). A total of 25 full-text articles were assessed for eligibility. Of these, three studies were excluded; detailed reasons for their exclusion are provided in the Appendix. Ultimately, 22 NMAs met the eligibility criteria [10,22-42].

The 22 included NMAs were published between 2019 and 2025 (Table 1). The majority (n=15, 68.2%) originated from China, with the remainder conducted in Spain and other regions. The scope of interventions was broad, encompassing various therapeutic and physical exercises (comparing up to 24 distinct modes), mind-body practices, and technology-assisted rehabilitation such as virtual reality and robotics. Included reviews synthesized data from 19 to 250 primary studies, representing total sample sizes of 811 to 13,011 participants. To document the specific primary data overlap across these reviews, we compiled a citation matrix (available in the Appendix).

**Table 1:** Study characteristics The symbol legend is as follows: ○ denotes that the item was available or reported, while ✕ denotes that the item was not available or reported. CRD: Centre for Reviews and Dissemination, CD: Cochrane Database, PRISMA NMA: Preferred Reporting Items for Systematic Reviews and Meta-analyses for network meta-analysis, RCTs: randomized controlled trials Shea et al. state that their methods section includes 'randomized and non-randomized controlled trials,' but the included studies in the main text consist only of randomized trials [16]. The authors used the Cochrane RoB-2 tool for assessment (→ the actual analysis is based on RCTs). Therefore, this table is presented in the format of "Eligibility/Actual included."

No	Author	Year	Country	Protocol Availability	PRISMA NMA Availability	Targeted Research	Number of papers, total sample size	Intervention
1	Chuang [22]	2022	Taiwan	○ CRD42020220521	PRISMA 2020 AMSTAR2	RCTs	23, 949	Modern technology (exergame and virtual reality)
2	Zhou [23]	2022	China	○ CRD42022324824	○	RCTs	20, 811	Aerobic and resistance training
3	Yang [10]	2022	China	○ CRD42021220052	○	RCTs (individual design, cluster design, or the first half of crossover)	250, 13011	Therapeutic exercises
4	Álvarez-Bueno [24]	2021	Spain	○ CRD42018087765	○	RCTs, non-RCTs(Eligibility) RCTs(Actual included)	56, 2740	Exercise
5	Wu [25]	2021	China	○ CRD42021224823	✕	RCTs	52, 1971	Aerobic exercise and mind-body exercise
6	Ernst [26]	2023	Germany	○ CD013856	○	RCTs	156, 7939	Physical exercise
7	Hao [27]	2022	China	✕	✕	RCTs	60, 2859	Ten different exercises
8	Lei [28]	2022	China	○ CRD42021285005	○	RCTs	20, 996	Tai chi exercises
9	Tang [29]	2019	China	✕	✕	－	19, 920	Exercise
10	Kwok [30]	2022	China	○ CRD42021226951	○	RCTs, crossover RCTs	37, 1454	Managing freezing of gait
11	Qin [31]	2021	China	✕	✕	RCTs	32, 1352	Multiple exercises
12	Mustafaoglu [32]	2022	Turkey	○ CRD42022301030	○	RCTs, crossover RCTs	60, 2037	Mind-body exercise
13	Qian [33]	2023	China	○ CRD42021220052	○	RCTs	199, 9523	24 exercise types
14	Lorenzo-Garcia [34]	2024	Spain	○ CRD42022351062	○	RCTs	86, 4,693	Sensorimotor training, and multiple exercises
15	Yau [35]	2024	Singapore	○ CRD42022301160	○	RCTs	51, 2,095	Internet-based, robotic, and Virtual reality
16	Lorenzo-García [36]	2023	Spain	○ CRD42021232911	○	RCTs	48, 2977	Multiple exercises
17	Zhang [37]	2023	China	○ CRD420212220052	○	RCTs	159, Not available	Multiple exercises
18	Wang [38]	2025	China	○ CRD42023422762	○	RCTs	81, 4,596	12 exercise types
19	Dou [39]	2025	China	○ CRD42024628687	○	RCTs	23, 1,330	Telemedicine interventions
20	Xie [40]	2025	China	○ CRD42024506919	○	RCTs	54, 2,828	12 exercise types
21	Fan [41]	2025	China	○ CRD42024517241	○	RCTs	73, 3,747	8 exercise types
22	Gao [42]	2024	China	○ CRD42022329780	○	RCTs	30, 2,264	Aerobic exercise, sensory exercise, mixed exercise

Methodological Quality (AMSTAR 2)

**Figure 2:**
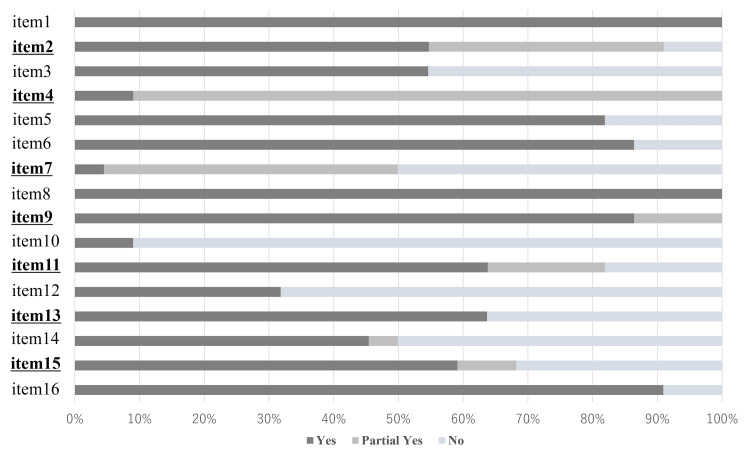
AMSTAR2 assessment Underlined text indicates critical domains

The AMSTAR 2 assessment revealed variable adherence to methodological standards (Figure 2, Table 2, and Table 3). While risk of bias assessment (Item 9) was a notable strength, fully met by approximately 86% of NMAs, overall confidence was severely limited by deficits in other critical domains. Notably, no NMA relied on updating search strategies from prior reviews. However, comprehensive literature searching (Item 4) and the reporting of excluded studies (Item 7) remained major stumbling blocks, with full adherence achieved in only 9.1% and 4.5% of reviews, respectively. Although protocol registration (Item 2) was established in over half of the reviews (54.5%), financial transparency (Item 10) was negligible, with fewer than 10% of reviews explicitly disclosing funding sources.

**Table 2:** Overall confidence rating in the results of the review

	Quality Assessment
High	No or one non-critical weakness: the systematic review provides an accurate and comprehensive summary of the results of the available studies that address the question of interest.
Moderate	More than one non-critical weakness: the systematic review has more than one weakness but no critical flaws. It may provide an accurate summary of the results of the available studies that were included in the review.
Low	One critical flaw with or without non-critical weaknesses: the review has a critical flaw and may not provide an accurate and comprehensive summary of the available studies that address the question of interest.
Critically low	More than one critical flaw with or without non-critical weaknesses: the review has more than one critical flaw and should not be relied on to provide an accurate and comprehensive summary of the available studies.

**Table 3:** The details of AMSTAR2 for all included studies Underlined text indicates critical domains, D: Domain

No	Author	D1	D2	D3	D4	D5	D6	D7	D8	D9	D10	D11	D12	D13	D14	D15	D16	Confidence
1	Chuang [22]	Y	PY	N	PY	N	N	PY	Y	Y	Y	PY	N	N	N	Y	Y	Low
2	Zhou [23]	Y	PY	N	PY	Y	Y	PY	Y	Y	N	PY	N	N	N	Y	Y	Low
3	Yang [10]	Y	PY	N	PY	Y	Y	PY	Y	PY	N	PY	Y	N	N	Y	Y	Low
4	Álvarez-Bueno [24]	Y	Y	N	PY	Y	Y	PY	Y	Y	N	Y	N	N	N	Y	Y	Low
5	Wu [25]	Y	PY	N	PY	N	Y	PY	Y	Y	N	Y	N	N	N	Y	Y	Low
6	Ernst [26]	Y	PY	N	Y	N	Y	Y	Y	Y	Y	Y	Y	Y	N	Y	Y	Moderate
7	Hao [27]	Y	N	N	PY	Y	N	PY	Y	Y	N	PY	N	N	N	Y	Y	Critically Low
8	Lei [28]	Y	PY	Y	PY	Y	Y	PY	Y	Y	N	N	N	N	N	Y	Y	Critically Low
9	Tang [29]	Y	N	N	PY	N	Y	N	Y	Y	N	N	N	N	N	N	Y	Critically Low
10	Kwok [30]	Y	Y	Y	Y	Y	Y	PY	Y	Y	N	Y	N	Y	Y	Y	Y	Moderate
11	Qin [31]	Y	Y	Y	PY	Y	N	PY	Y	Y	N	N	N	Y	N	N	N	Critically Low
12	Mustafaoglu [32]	Y	PY	Y	PY	Y	Y	PY	PY	Y	N	Y	N	Y	Y	Y	Y	Moderate
13	Qian [33]	Y	Y	Y	PY	Y	Y	N	Y	PY	N	Y	Y	Y	Y	Y	Y	Low
14	Lorenzo-Garcia [34]	Y	Y	Y	PY	Y	Y	N	Y	Y	N	Y	Y	Y	Y	PY	Y	Low
15	Yau [35]	Y	Y	Y	PY	Y	Y	N	Y	Y	N	Y	N	Y	Y	Y	Y	Low
16	Lorenzo-García [36]	Y	Y	Y	PY	Y	Y	N	Y	Y	N	Y	N	Y	Y	Y	Y	Low
17	Zhang [37]	Y	Y	Y	PY	Y	Y	N	Y	PY	N	Y	N	Y	Y	N	Y	Critically Low
18	Wang [38]	Y	Y	Y	PY	Y	Y	N	Y	Y	N	Y	N	Y	Y	N	Y	Critically Low
19	Dou [39]	Y	Y	Y	PY	Y	Y	N	Y	Y	N	Y	Y	Y	Y	Y	Y	Low
20	Xie [40]	Y	Y	Y	PY	Y	Y	N	Y	Y	N	Y	N	Y	Y	N	Y	Critically Low
21	Fan [41]	Y	Y	Y	PY	Y	Y	N	Y	Y	N	Y	Y	Y	Y	N	Y	Critically Low
22	Gao [42]	Y	PY	N	PY	Y	Y	PY	Y	Y	N	PY	Y	N	PY	PY	Y	Low

Stratifying these ratings by geographic origin (China (n=15) vs. other regions (n=7)) revealed largely consistent methodological profiles (see the Appendix). Deficiencies in critical domains, such as comprehensive searching, were universal across regions, suggesting that these shortcomings reflect field-wide challenges rather than region-specific reporting biases. Based on these assessments, no NMA was rated as “High” confidence, and only three achieved a “Moderate” rating.

Reporting Quality (PRISMA-NMA)

**Figure 3:**
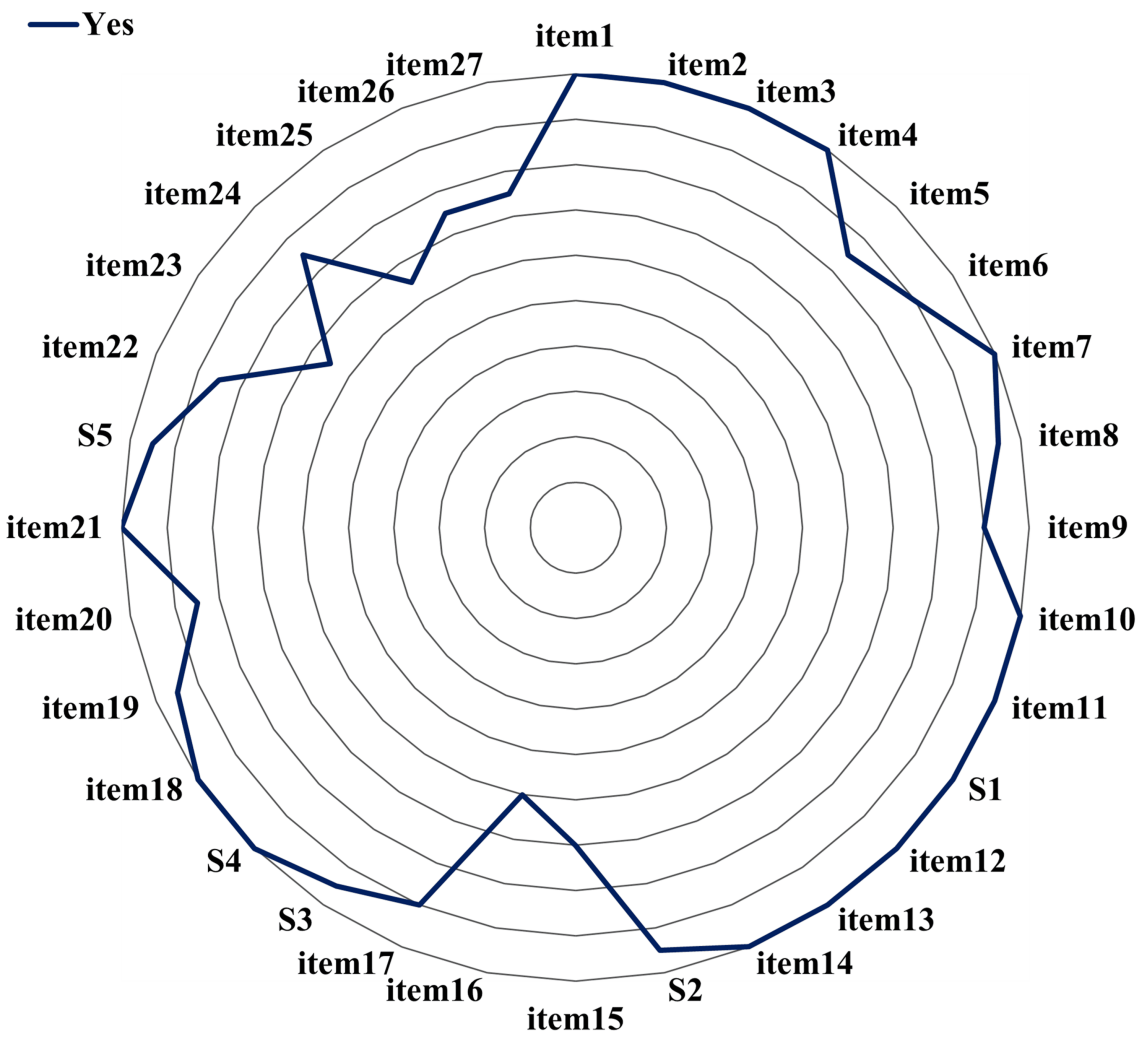
PRISMA-NMA assessment PRISMA: Preferred Reporting Items for Systematic Reviews and Meta-Analyses; NMA: network meta-analysis

Adherence to PRISMA-NMA reporting standards was generally satisfactory (Figure 3, Table 4). NMA-specific items (S1-S5), such as network geometry and inconsistency checks, were reported in 95% or more of the studies, indicating a solid grasp of core NMA reporting requirements. However, a sensitivity analysis revealed that checklist submission was not a definitive proxy for quality (Appendix). While the group submitting checklists (n=8) showed higher adherence to protocol registration, they performed no better than the non-checklist group in reporting funding sources.

**Table 4:** The details of PRISMA-NMA for all included studies Y: Yes, N: No PRISMA: Preferred Reporting Items for Systematic Reviews and Meta-Analyses; NMA: network meta-analysis

No	Author	1	2	3	4	5	6	7	8	9	10	11	S1	12	13	14	S2	15	16	17	S3	S4	18	19	20	21	S5	22	23	24	25	26	27
1	Chuang [22]	Y	Y	Y	Y	Y	Y	Y	Y	Y	Y	Y	Y	Y	Y	Y	Y	Y	Y	Y	Y	Y	Y	Y	Y	Y	Y	Y	N	Y	Y	Y	Y
2	Zhou [23]	Y	Y	Y	Y	Y	Y	Y	Y	Y	Y	Y	Y	Y	Y	Y	Y	Y	N	Y	Y	Y	Y	Y	Y	Y	Y	Y	N	Y	Y	Y	Y
3	Yang [10]	Y	Y	Y	Y	Y	Y	Y	Y	Y	Y	Y	Y	Y	Y	Y	Y	Y	Y	Y	Y	Y	Y	Y	Y	Y	Y	Y	Y	Y	Y	Y	Y
4	Álvarez-Bueno [24]	Y	Y	Y	Y	Y	Y	Y	Y	Y	Y	Y	Y	Y	Y	Y	Y	Y	Y	Y	Y	Y	Y	Y	Y	Y	Y	Y	Y	Y	Y	Y	Y
5	Wu [25]	Y	Y	Y	Y	Y	Y	Y	Y	Y	Y	Y	Y	Y	Y	Y	Y	Y	Y	Y	Y	Y	Y	Y	Y	Y	Y	Y	Y	Y	Y	Y	Y
6	Ernst [26]	Y	Y	Y	Y	Y	Y	Y	Y	Y	Y	Y	Y	Y	Y	Y	Y	Y	Y	Y	Y	Y	Y	Y	Y	Y	Y	Y	Y	Y	Y	Y	Y
7	Hao [27]	Y	Y	Y	Y	N	Y	Y	Y	Y	Y	Y	Y	Y	Y	Y	Y	Y	N	Y	Y	Y	Y	Y	N	Y	Y	Y	N	Y	Y	Y	Y
8	Lei [28]	Y	Y	Y	Y	Y	Y	Y	Y	Y	Y	Y	Y	Y	Y	Y	N	Y	N	Y	Y	Y	Y	Y	Y	Y	N	Y	N	Y	Y	Y	Y
9	Tang [29]	Y	Y	Y	Y	N	Y	Y	Y	N	Y	Y	Y	Y	Y	Y	Y	N	N	Y	Y	Y	Y	Y	Y	Y	Y	N	N	Y	Y	Y	Y
10	Kwok [30]	Y	Y	Y	Y	Y	Y	Y	Y	Y	Y	Y	Y	Y	Y	Y	Y	Y	N	Y	Y	Y	Y	Y	Y	Y	Y	Y	Y	Y	Y	Y	Y
11	Qin [31]	Y	Y	Y	Y	N	Y	Y	Y	Y	Y	Y	Y	Y	Y	Y	Y	N	N	Y	Y	Y	Y	Y	N	Y	Y	N	N	Y	Y	Y	N
12	Mustafaoglu [32]	Y	Y	Y	Y	Y	Y	Y	Y	Y	Y	Y	Y	Y	Y	Y	Y	Y	Y	N	Y	Y	Y	Y	Y	Y	Y	Y	Y	Y	N	Y	Y
13	Qian [33]	Y	Y	Y	Y	Y	Y	Y	Y	Y	Y	Y	Y	Y	Y	Y	Y	Y	Y	Y	Y	Y	Y	Y	Y	Y	Y	Y	Y	Y	Y	Y	Y
14	Lorenzo-Garcia [34]	Y	Y	Y	Y	Y	Y	Y	Y	Y	Y	Y	Y	Y	Y	Y	Y	Y	Y	Y	Y	Y	Y	Y	N	Y	Y	Y	Y	Y	Y	Y	N
15	Yau [35]	Y	Y	Y	Y	Y	Y	Y	Y	Y	Y	Y	Y	Y	Y	Y	Y	N	N	N	Y	Y	Y	Y	Y	Y	Y	N	Y	N	N	N	Y
16	Lorenzo-García [36]	Y	Y	Y	Y	Y	Y	Y	Y	Y	Y	Y	Y	Y	Y	Y	Y	Y	Y	Y	Y	Y	Y	Y	Y	Y	Y	Y	Y	N	Y	Y	Y
17	Zhang [37]	Y	Y	Y	Y	Y	N	Y	Y	Y	Y	Y	Y	Y	Y	Y	Y	N	Y	N	Y	Y	Y	Y	Y	Y	Y	Y	Y	Y	N	N	Y
18	Wang [38]	Y	Y	Y	Y	Y	N	Y	Y	Y	Y	Y	Y	Y	Y	Y	Y	N	N	Y	Y	Y	Y	Y	Y	Y	Y	Y	N	N	N	N	Y
19	Dou [39]	Y	Y	Y	Y	Y	Y	Y	Y	Y	Y	Y	Y	Y	Y	Y	Y	N	Y	Y	N	Y	Y	Y	Y	Y	Y	Y	Y	Y	N	N	Y
20	Xie [40]	Y	Y	Y	Y	Y	Y	Y	Y	Y	Y	Y	Y	Y	Y	Y	Y	Y	Y	Y	Y	Y	Y	Y	Y	Y	Y	Y	Y	Y	N	Y	N
21	Fan [41]	Y	Y	Y	Y	Y	Y	Y	Y	N	Y	Y	Y	Y	Y	Y	Y	Y	Y	Y	Y	Y	Y	Y	Y	Y	Y	Y	Y	Y	N	N	N
22	Gao [42]	Y	Y	Y	Y	Y	Y	Y	Y	Y	Y	Y	Y	Y	Y	Y	Y	Y	Y	Y	Y	Y	Y	Y	Y	Y	Y	Y	Y	Y	N	Y	N

Additional Analyses and Synthesis

As outlined in Table 5, robust testing was uneven. Assessment of small-study effects was common (72.7%), but sensitivity analyses and subgroup analyses were underutilized, the latter appearing in only two reviews (9.1%). Detailed domain-level adherence breakdown is available in the Appendix. Finally, Table 6 juxtaposes the key clinical conclusions against their methodological ratings. This synthesis highlights that while many studies offer definitive therapeutic recommendations, these claims are frequently underpinned by evidence of low to critically low methodological confidence.

**Table 5:** Overview of sensitivity, subgroup, meta-regression, and small-study effect analyses performed in the included NMAs NMA: Network meta-analysis

Study	Sensitivity analysis	Subgroup analysis	Meta-regression	Small-study effects
Chuang 2022 [22]	No	No	No	Yes
Zhou 2022 [23]	No	No	No	Yes
Yang 2022 [10]	Yes	No	Yes	Yes
Alvarez-Bueno 2021 [24]	Yes	No	Yes	Yes
Wu 2021 [25]	Yes	No	Yes	Yes
Ernst 2023 [26]	Yes	Yes	No	Yes
Hao 2022 [27]	No	No	No	Yes
Lei 2022 [28]	No	No	No	Yes
Tang 2019 [29]	No	No	No	Yes
Kwok 2022 [30]	No	Yes	Yes	Yes
Li 2021 [31]	No	No	No	No
Mustafaoglu 2022 [32]	No	No	No	Yes
Qian 2023 [33]	Yes	No	Yes	Yes
Lorenzo-García 2024 [34]	Yes	No	No	Yes
Yau 2024 [35]	Yes	No	No	Yes
Lorenzo-García 2023 [36]	Yes	No	No	Yes
Zhang 2023 [37]	No	No	No	No
Wang 2025 [38]	No	No	Yes	No
Dou 2025 [39]	Yes	No	Yes	Yes
Xie 2025 [40]	No	No	Yes	No
Fan 2025 [41]	No	No	Yes	No
Gao 2024 [42]	Yes	No	Yes	Yes

Discussion

For clinicians and patients seeking the "optimal" exercise intervention for PD, the findings of this review suggest a need for significant caution. While the volume of NMAs has surged, predominantly driven by research groups in China, the certainty of evidence underpinning their therapeutic recommendations is often fragile. Specifically, our assessment revealed that definitive clinical conclusions are frequently derived from studies with "Low" or "Critically Low" methodological confidence. This disconnect implies that current treatment rankings may not reliably reflect true comparative efficacy, posing a real risk to evidence-based clinical decision-making [43].

The primary threat to validity stems from foundational methodological deficits rather than reporting style. While reporting quality (PRISMA-NMA) appeared generally satisfactory, this often masked deeper flaws in study conduct (AMSTAR 2). Crucially, comprehensive literature searching and the justification for excluded studies were major stumbling blocks. These are not merely bureaucratic omissions; the failure to rigorously search for and document all relevant evidence increases the risk of selection bias, potentially distorting the estimated treatment effects [14,21,44,45]. Furthermore, although protocol registration was present in over half the reviews, financial transparency was negligible across the board. This lack of disclosure regarding funding sources represents a significant blind spot that hinders the assessment of potential conflicts of interest [46]. Importantly, the problem of suboptimal NMA quality is not confined to PD [14,47,48]. Previous meta-epidemiological studies have reported similar shortcomings across various medical fields, indicating that these are systemic issues in the production of evidence.

Our sensitivity analysis addressed potential concerns regarding the geographic concentration of research. Although over two-thirds of the included NMAs originated from China, we found no systematic methodological deviation between Chinese and non-Chinese reviews (see the Appendix). Common strengths (e.g., risk of bias assessment) and weaknesses (e.g., search strategies) were consistent across regions. This suggests that the identified shortcomings reflect field-wide challenges in the conduct of NMAs rather than region-specific reporting biases. Therefore, the results of this review are generalizable to the broader landscape of exercise therapy research.

A discrepancy between "reporting" and "methodological rigor" was a key finding. Many studies achieved high PRISMA-NMA scores, suggesting authors know how to report a meta-analysis, yet failed critical AMSTAR 2 domains, indicating they may not know how to conduct one rigorously. For instance, checklist submission improved protocol registration but did not guarantee better transparency in funding disclosure. Consequently, checklist adherence alone serves as an insufficient proxy for study quality. Clinicians should look beyond the summary of findings and critically appraise the underlying methods, particularly whether the search was comprehensive and if exclusion reasons were transparent.

To improve the quality of future NMAs and ensure they provide actionable evidence, researchers must prioritize methodological rigor over mere output. This requires: (i) mandatory pre-registration of protocols with detailed analysis plans [49]; (ii) reproducible and comprehensive search strategies that update rather than recycle previous efforts; and (iii) transparent reporting of all excluded studies and funding sources. Additionally, future reviews should explicitly evaluate the certainty of evidence using frameworks like GRADE or CINeMA to help clinicians distinguish between robust findings and those requiring caution [50]. Furthermore, the scarcity of subgroup and sensitivity analyses identified in this review highlights a need to better explore heterogeneity. Future NMAs should rigorously test whether exercise benefits remain consistent across different patient characteristics to support personalized rehabilitation strategies [51].

Limitations

This review has limitations. First, the sample size (n=22) is relatively small, though it captures the current state of NMAs in this specific domain. Second, we assessed reporting and methodological quality but did not re-analyze the primary data to verify the effect sizes of the exercise interventions. Future research should focus on conducting high-quality, living NMAs [52] that address these deficiencies to support a dynamic and individualized evidence base for optimizing exercise therapy in PD.

## Conclusions

While NMAs of exercise for PD are proliferating, their methodological quality currently lags their clinical ambition. No study achieved a high confidence rating, and the widespread lack of comprehensive searching and financial transparency undermines the reliability of their conclusions. Moving forward, the research community must shift focus from quantity to quality. By adhering to established conduct standards, not just reporting checklists, future NMAs can provide the robust evidence needed to truly optimize exercise prescriptions for people with PD.

## Figures and Tables

**Figure 4:**
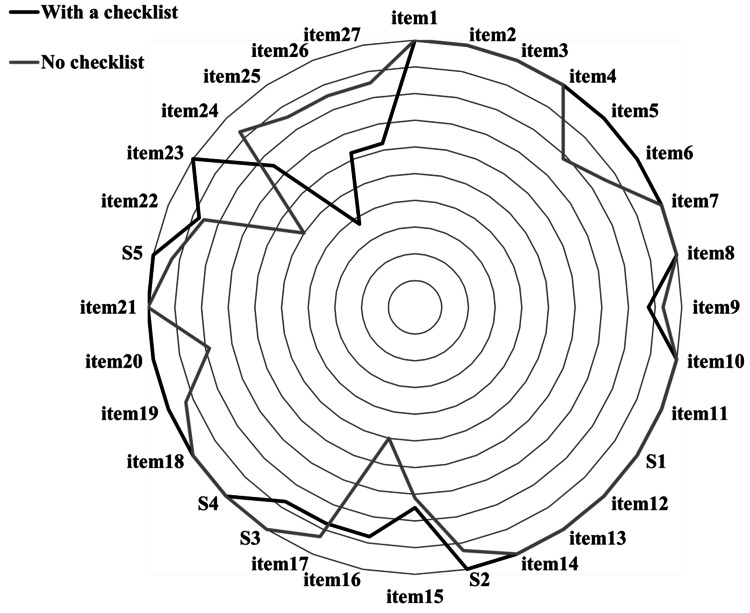
Comparison of PRISMA-NMA reporting adherence between NMAs with and without submitted checklists PRISMA: Preferred Reporting Items for Systematic Reviews and Meta-Analyses; NMA: network meta-analysis

**Figure 5:**
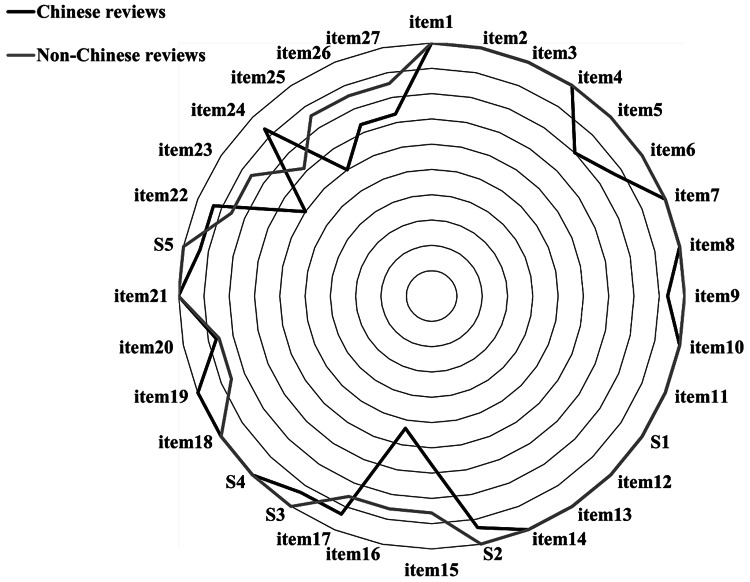
Comparison of PRISMA-NMA reporting adherence between Chinese and non-Chinese research groups PRISMA: Preferred Reporting Items for Systematic Reviews and Meta-Analyses; NMA: network meta-analysis

## Appendices

Search strategy

PubMed

#1. ("Physical Therapy Modalities"[MeSH Terms] OR "Virtual Reality"[MeSH Terms] OR "exercise"[MeSH Terms] OR "exercis*"[Title/Abstract] OR ("aerobic exercise"[All Fields] OR "aquatic exercise"[All Fields] OR "balance training"[All Fields] OR "body weight support treadmill"[All Fields] OR "gait training"[All Fields] OR "high-speed resistance training"[All Fields] OR "multicomponent exercise program"[All Fields] OR "multidisciplinary exercise program"[All Fields] OR "Nordic Walking"[All Fields] OR ("Physical Therapy Modalities"[MeSH Terms] OR ("physical"[All Fields] AND "therapy"[All Fields] AND "modalities"[All Fields]) OR "Physical Therapy Modalities"[All Fields] OR "physiotherapies"[All Fields] OR "physiotherapy"[All Fields]) OR ("pilate"[All Fields] OR "pilates"[All Fields]) OR "power training"[All Fields] OR "Robotic-assisted gait training"[All Fields] OR ("stretch"[All Fields] OR "stretched"[All Fields] OR "stretches"[All Fields] OR "stretching"[All Fields] OR "stretchings"[All Fields]) OR ("tango"[All Fields] OR "tangos"[All Fields]) OR "treadmill training"[All Fields] OR "walking"[All Fields] OR "whole body vibration"[All Fields]))

#2. ("parkinsonian disorders"[MeSH Terms] OR "Parkinson Disease"[MeSH Terms] OR "parkinson's disease"[Title/Abstract] OR "parkinsonian disorders"[Title/Abstract] OR "parkinsonism"[Title/Abstract] OR "parkinson"[Title/Abstract] OR "Parkinson Disease"[Title/Abstract] OR "parkinsons disease"[Title/Abstract] OR "idiopathic parkinson disease"[Title/Abstract] OR "idiopathic parkinsons disease"[Title/Abstract] OR "idiopathic parkinson s disease"[Title/Abstract])

#3. ("network meta analysis"[MeSH Terms] OR "mixed-treatment comparisons meta-analysis"[All Fields] OR "multiple treatments meta-analysis"[All Fields] OR "multiple treatmen

t comparison"[All Fields] OR "network meta analysis"[All Fields] OR "indirect comparison"[All Fields]) OR (("network*"[All Fields] AND "adj3"[All Fields] AND ("meta analy*"[All Fields] OR "metanaly*"[All Fields] OR "metaanaly*"[All Fields] OR ("met"[All Fields] AND "analy*"[All Fields])) AND ("meta analy*"[All Fields] OR "metanaly*"[All Fields] OR "metaanaly*"[All Fields] OR ("met"[All Fields] AND "analy*"[All Fields])) AND "mp"[All Fields]) OR ("indirect*"[All Fields] AND "adj"[All Fields] AND "comparison*"[All Fields]) OR (("mixed"[All Fields] OR "mixes"[All Fields] OR "mixing"[All Fields] OR "mixings"[All Fields]) AND "treatment*"[All Fields] AND "adj"[All Fields] AND "comparison*"[All Fields]) OR (("multiple"[All Fields] OR "multiples"[All Fields]) AND "treatment*"[All Fields] AND "adj"[All Fields] AND "comparison*"[All Fields]) OR ("multi treatment*"[All Fields] AND "adj"[All Fields] AND "comparison*"[All Fields]) OR "comparison*"[All Fields] OR (("mixed"[All Fields] OR "mixes"[All Fields] OR "mixing"[All Fields] OR "mixings"[All Fields]) AND "comparison*"[All Fields]))  490

#4. #1 AND #2 AND #3

*CENTRAL*　

#1. MeSH descriptor: [Parkinsonian Disorders] explode all trees 

#2. parkinson*

#3. (aerobic exercise or aquatic exercise or balance training or body weight support treadmill or Dance Therapy or exercise* or Exercise Movement Techniques or gait training or high-speed resistance training or hydrotherapy or multicomponent exercise program or multidisciplinary exercise program or Nordic Walking or Physiotherapy or pilates or power training or Qigong or resistance training or Robotic-assisted gait training or stretch or tai ji or Tango or treadmill training or walking or Virtual Reality or whole body vibration or Yoga) in Trials (Word variations have been searched) 

#4. MeSH descriptor: [resistance training] explode all trees 

#5. MeSH descriptor: [exercise] explode all trees　

#6. MeSH descriptor: [tai ji] explode all trees 

#7. MeSH descriptor: [Qigong] explode all trees 

#8. MeSH descriptor: [Exercise Movement Technique] explode all trees 

#9. MeSH descriptor: [Yoga] explode all trees 

#10. MeSH descriptor: [Virtual Reality] explode all trees 

#11. MeSH descriptor: [hydrotherapy] explode all trees 

#12. MeSH descriptor: [Dance Therapy] explode all trees 

#13. #1 or #2 

#14. #3 or #4 or #5 or #6 or #7 or #8 or #9 or #10 or #11 or #12 

#15. [mh "network meta analysis"] OR "mixed-treatment comparisons meta-analysis" OR "multiple treatments meta-analysis" OR "multiple treatment comparison" OR "network meta analysis" OR "indirect comparison" OR (network* AND adj3 AND (("meta" NEXT analy*) OR metanaly* OR metaanaly* OR (met AND analy*)) AND (("meta" NEXT analy*) OR metanaly* OR metaanaly* OR (met AND analy*)) AND mp) OR (indirect* AND adj AND comparison*) OR ((mixed OR mixes OR mixing OR mixings) AND treatment* AND adj AND comparison*) OR ((multiple OR multiples) AND treatment* AND adj AND comparison*) OR (("multi" NEXT treatment*) AND adj AND comparison*) OR comparison* OR ((mixed OR mixes OR mixing OR mixings) AND comparison*)) 

#13 AND #14 AND #15  

*CINAHL* 　

#1. MH"parkinson disease" OR parkinsonian disorders OR parkinsonism OR parkinson OR parkinson disease OR parkinsons disease OR parkinson’s disease OR idiopathic parkinson disease OR idiopathic parkinsons disease OR idiopathic parkinson’s disease

#2. (aerobic exercise or aquatic exercise or balance training or body weight support treadmill or Dance Therapy or exercise* or Exercise Movement Techniques or gait training or high-speed resistance training or hydrotherapy or multicomponent exercise program or multidisciplinary exercise program or Nordic Walking or Physiotherapy or pilates or power training or Qigong or resistance training or Robotic-assisted gait training or stretch or tai ji or Tango or treadmill training or walking or Virtual Reality or whole body vibration or Yoga) in Trials (Word variations have been searched)

#3. MH”resistance training”

#4. MH”exercise”

#5. MH”tai ji”

#6. MH”Qigong”

#7. MH”Exercise Movement Technique”

#8. MH”Yoga”

#9. MH”Virtual”

#10. MH”hydrotherapy”

#11. MH”Dance Therapy”

#12. #2 or #3 or #4 or #5 or #6 or #7 or #8 or #9 or #10 or #11

#13. (MH "network meta analysis+") OR "mixed-treatment comparisons meta-analysis" OR "multiple treatments meta-analysis" OR "multiple treatment comparison" OR "network meta analysis" OR "indirect comparison" OR (network* AND adj3 AND ("meta analy*" OR metanaly* OR metaanaly* OR (met AND analy*)) AND ("meta analy*" OR metanaly* OR metaanaly* OR (met AND analy*)) AND mp) OR (indirect* AND adj AND comparison*) OR ((mixed OR mixes OR mixing OR mixings) AND treatment* AND adj AND comparison*) OR ((multiple OR multiples) AND treatment* AND adj AND comparison*) OR ("multi treatment*" AND adj AND comparison*) OR comparison* OR ((mixed OR mixes OR mixing OR mixings) AND comparison*)

*Web of Science* 　

#1. TOPIC: ("Idiopathic Parkinson's Disease" or "Lewy Body Parkinson's Disease" or "Parkinson's Disease, Idiopathic" or "Parkinson's Disease, Lewy Body" or "Parkinson Disease, Idiopathic" or "Parkinson's Disease" or "Idiopathic Parkinson Disease" or "Lewy Body Parkinson Disease" or "Primary Parkinsonism" or "Parkinsonism, Primary" or "Paralysis Agitans") 

Indexes=SCI-EXPANDED, SSCI, A&HCI, CPCI-S, CPCI-SSH, BKCI-S, BKCI-SSH, ESCI, CCR-EXPANDED, IC Timespan=All years

#2. TOPIC: (“aerobic exercise” or “aquatic exercise” or “balance training” or “body weight support treadmill” or “Dance Therapy or exercise*” or “Exercise Movement Techniques” or “gait training” or “high-speed resistance training” or “hydrotherapy” or “multicomponent exercise program” or “multidisciplinary exercise program” or “Nordic Walking” or “Physiotherapy” or pilates or “power training” or Qigong or “resistance training” or “Robotic-assisted gait training” or stretch or “tai ji” or Tango or “treadmill training” or “walking” or “Virtual Reality” or “whole body vibration” or Yoga) 

Indexes=SCI-EXPANDED, SSCI, A&HCI, CPCI-S, CPCI-SSH, BKCI-S, BKCI-SSH, ESCI, CCR-EXPANDED, IC Timespan=All years

#3. TOPIC: ("Resistance training” or “Training, Resistance” or “Strength Training” or “Training, Strength” or “Weight-Lifting Strengthening Program” or “Strengthening Program, Weight-Lifting” or “Strengthening Programs, Weight-Lifting” or “Weight Lifting Strengthening Program” or “Weight-Lifting Strengthening Programs” or “Weight-Lifting Exercise Program” or “Exercise Program, Weight-Lifting” or “Exercise Programs, Weight-Lifting” or “Weight Lifting Exercise Program” or “Weight-Lifting Exercise Programs” or “Weight-Bearing Strengthening Program” or “Strengthening Program, Weight-Bearing” or “Strengthening Programs, Weight-Bearing” or “Weight Bearing Strengthening Program” or “Weight-Bearing Strengthening Programs” or “Weight-Bearing Exercise Program”) 

Indexes=SCI-EXPANDED, SSCI, A&HCI, CPCI-S, CPCI-SSH, BKCI-S, BKCI-SSH, ESCI, CCR-EXPANDED, IC Timespan=All years

#4. TOPIC: (Exercise* or “Exercise Program, Weight-Bearing” or “Exercise Programs, Weight-Bearing” or “Weight Bearing Exercise Program” or “Weight-Bearing Exercise Programs” Exercise* or “Physical Activity” or “Activities, Physical” or “Activity, Physical” or “Physical Activities” or “Exercise, Physical” or “Exercises, Physical” or “Physical Exercise” or “Physical Exercises” or “Exercise, Isometric” or “Exercises, Isometric” or “Isometric Exercises” or “Isometric Exercise” or “Exercise, Aerobic” or “Aerobic Exercise” or “Aerobic Exercises” or “Exercises, Aerobic” or “Exercise Training” or “Exercise Trainings” or “Training, Exercise” or “Trainings, Exercise”) 

Indexes=SCI-EXPANDED, SSCI, A&HCI, CPCI-S, CPCI-SSH, BKCI-S, BKCI-SSH, ESCI, CCR-EXPANDED, IC Timespan=All years

#5 TOPIC: (“Tai-ji” or “Tai Chi” or “Chi, Tai” or “Tai Ji Quan” or “Ji Quan, Tai” or “Quan, Tai Ji” or Taiji or Taijiquan or “T'ai Chi” or “Tai Chi Chuan” Qigong or “Qi Gong” or “Ch'i Kung”) 

Indexes=SCI-EXPANDED, SSCI, A&HCI, CPCI-S, CPCI-SSH, BKCI-S, BKCI-SSH, ESCI, CCR-EXPANDED, IC Timespan=All years

#6. TOPIC: (("Exercise Movement Techniques" or "Movement Techniques, Exercise" or "Exercise Movement Technics" or "Pilates-Based Exercises" or "Exercises, Pilates-Based" or "Pilates Based Exercises" or "Pilates Training" or "Training, Pilates"）) 

Indexes=SCI-EXPANDED, SSCI, A&HCI, CPCI-S, CPCI-SSH, BKCI-S, BKCI-SSH, ESCI, CCR-EXPANDED, IC Timespan=All years

#7. TOPIC: (("Virtual Reality" or "Reality, Virtual" or "Virtual Reality, Educational" or "Educational Virtual Realities" or "Educational Virtual Reality" or "Reality, Educational Virtual" or "Virtual Realities, Educational" or "Virtual Reality, Instructional" or "Instructional Virtual Realities" or "Instructional Virtual Reality" or "Realities, Instructional Virtual" or "Reality, Instructional Virtual" or "Virtual Realities, Instructional") ) 

Indexes=SCI-EXPANDED, SSCI, A&HCI, CPCI-S, CPCI-SSH, BKCI-S, BKCI-SSH, ESCI, CCR-EXPANDED, IC Timespan=All years

#8. TOPIC: ((hydrotherapy or Hydrotherapies or “Whirlpool Baths” or “Bath, Whirlpool” or “Baths, Whirlpool” or “Whirlpool Bath”) ) 

Indexes=SCI-EXPANDED, SSCI, A&HCI, CPCI-S, CPCI-SSH, BKCI-S, BKCI-SSH, ESCI, CCR-EXPANDED, IC Timespan=All years

#9. TOPIC: ((“Dance Therapy” or “Therapy, Dance” or “Dance Therapies” or “Therapies, Dance”) ) 

Indexes=SCI-EXPANDED, SSCI, A&HCI, CPCI-S, CPCI-SSH, BKCI-S, BKCI-SSH, ESCI, CCR-EXPANDED, IC Timespan=All years

#10. TOPIC: ((Yoga or “Muscle Stretching Exercises”) ) 

Indexes=SCI-EXPANDED, SSCI, A&HCI, CPCI-S, CPCI-SSH, BKCI-S, BKCI-SSH, ESCI, CCR-EXPANDED, IC Timespan=All years

#11. #2 OR #3 OR #4 OR #5 OR #6 OR #7 OR #8 OR #9 OR #10

#12. TOPIC: ("network meta analysis" OR "mixed-treatment comparisons meta-analysis" OR "multiple treatments meta-analysis" OR "multiple treatment comparison" OR "network meta analysis" OR "indirect comparison") OR (network* AND “adj3” AND ("meta analy*" OR metanaly* OR metaanaly* OR (met AND analy*)) AND ("meta analy*" OR metanaly* OR metaanaly* OR (met AND analy*)) AND mp OR (indirect* AND “adj” AND comparison*) OR ((mixed OR mixes OR mixing OR mixings) AND treatment* AND “adj” AND comparison*) OR ((multiple OR multiples) AND treatment* AND “adj” AND comparison*) OR ("multi treatment*" AND “adj” AND comparison*) OR comparison* OR ((mixed OR mixes OR mixing OR mixings) AND comparison*))

Indexes=SCI-EXPANDED, SSCI, A&HCI, CPCI-S, CPCI-SSH, BKCI-S, BKCI-SSH, ESCI, CCR-EXPANDED, IC Timespan=All years#13. #1 AND #11 AND #12

*Clinical Trials.gov*　

Condition or disease. Parkinson’s disease

Other terms. Network meta analysis 

*PEDro*　

Abstract & Title: (parkinson's disease) (network meta-analysis)

Method: systematic review  

*Epistemonikos*　

(title:((title:("parkinson's disease") OR abstract:("parkinson's disease")) AND (title:("network meta-analysis" OR  "network meta analysis" OR "mixed-treatment comparisons meta-analysis" OR "multiple treatments meta-analysis" OR "multiple treatment comparison" OR "indirect comparison") OR abstract:("network meta-analysis" OR  "network meta analysis" OR "mixed-treatment comparisons meta-analysis" OR "multiple treatments meta-analysis" OR "multiple treatment comparison" OR "indirect comparison"))) OR abstract:((title:("parkinson's disease") OR abstract:("parkinson's disease")) AND (title:("network meta-analysis" OR  "network meta analysis" OR "mixed-treatment comparisons meta-analysis" OR "multiple treatments meta-analysis" OR "multiple treatment comparison" OR "indirect comparison") OR abstract:("network meta-analysis" OR  "network meta analysis" OR "mixed-treatment comparisons meta-analysis" OR "multiple treatments meta-analysis" OR "multiple treatment comparison" OR "indirect comparison"))))

**Table 6:** List of excluded studies with reasons

No	Title	Author	Year	Country	Reason for exclusion
1	Efficacy of non-pharmacological interventions for depression in individuals with Parkinson's disease: A systematic review and network meta-analysis	Yuxin Wang	2022	China	Wrong intervention As it includes surgical treatments and non-invasive brain stimulation therapies
2	Nintendo WiiTM versus Xbox KinectTM for functional locomotion in people with Parkinson’s disease: a systematic review and network meta-analysis	Nicola Marotta	2020	Italy	Wrong study design Since the number of nodes is 3 (the protocol is set to exclude 4 or fewer nodes), this setting is invalid.
3	Interventions to improve gait in Parkinson's disease: a systematic review of randomized controlled trials and network meta-analysis	Victor Schwartz Hvingelby	2022	Denmark	Wrong intervention As it includes surgical treatments and non-invasive brain stimulation therapies

**Table 7:** Citation matrix mapping primary randomized controlled trials to the included network meta-analyses ACTR: Australian Clinical Trials Registry; ACTRN: Australian New Zealand Clinical Trials Registry Number; CEP: Comitê de Ética em Pesquisa (Research Ethics Committee); IRCT: Iranian Registry of Clinical Trials; ISRCTN: International Standard Randomised Controlled Trial Number; N/A: Not Available; NCT: National Clinical Trial (ClinicalTrials.gov identifier); NTR: Netherlands Trial Register.

Study	Title	Trial registration	Reference number of the cited systematic review
Amano et al., 2013	The effect of Tai Chi exercise on gait initiation and gait performance in persons with Parkinson's disease	NCT00611481	[26], [37]
Arfa-Fatollahkhani et al., 2019	Effects of treadmill training on the balance, functional capacity and quality of life in Parkinson’s disease: A randomized clinical trial	IRCT2015062322891N1	[26], [34], [37]
Capato et al., 2020	Multimodal Balance Training Supported by Rhythmic Auditory Stimuli in Parkinson Disease: Effects in Freezers and Nonfreezers	NCT02488265	[34], [37]
Canning et al., 2012	Home-based treadmill training for individuals with Parkinson's disease: a randomized controlled pilot trial	NCT00261781	[23], [26], [42]
Carvalho et al., 2015	Comparison of strength training, aerobic training, and additional physical therapy as supplementary treatments for Parkinson's disease: pilot study	008-09-CEP	[23], [26]
Cugusi et al., 2015	Effects of a Nordic walking program on motor and non-motor symptoms, functional performance and body composition in patients with Parkinson's disease	N/A	[23], [26]
Dibble et al., 2006	High-intensity resistance training amplifies muscle hypertrophy and functional gains in persons with Parkinson's disease	N/A	[23], [37]
Dibble et al., 2015	Exercise and medication effects on persons with Parkinson disease across the domains of disability: a randomized clinical trial	N/A	[23], [37]
Fisher et al., 2008	The effect of exercise training in improving motor performance and corticomotor excitability in people with early Parkinson's disease	N/A	[23], [26], [37]
Gao et al., 2014	Effects of tai chi on balance and fall prevention in Parkinson’s disease: a randomized controlled trial	N/A	[26], [34]
Goodwin et al., 2011	An exercise intervention to prevent falls in people with Parkinson's disease: a pragmatic randomised controlled trial	ISRCTN50793425	[26], [42]
Hackney et al., 2009	Effects of dance on movement control in Parkinson's disease: a comparison of Argentine tango and American ballroom	N/A	[26], [31], [37]
Hackney et al., 2007	Effects of tango on functional mobility in Parkinson's disease: a preliminary study	ACTRN12606000344583	[26], [37]
Kurtais et al., 2008	Does treadmill training improve lower-extremity tasks in Parkinson disease? A randomized controlled trial	N/A	[23], [26], [31], [37]
Miyai et al., 2002	Long-term effect of body weight-supported treadmill training in Parkinson's disease: a randomized controlled trial	N/A	[26], [37]
Morris et al., 2009	A randomized controlled trial of movement strategies compared with exercise for people with Parkinson's disease	ACTR 8221	[26], [37]
Morris et al., 2017	Home based exercises to reduce falls in people with Parkinson's disease: a randomized trial	N/A	[26], [42]
Nieuwboer et al., 2007	Cueing training in the home improves gait‐related mobility in Parkinson's disease: the RESCUE trial	N/A	[26], [37], [42]
Picelli et al., 2016	Effects of treadmill training on cognitive and motor features of patients with mild to moderate Parkinson's disease: a pilot, single-blind, randomized controlled trial	NCT02028861	[26], [31], [37]
Pohl et al., 2013	The Ronnie Gardiner Rhythm and Music Method - a feasibility study in Parkinson's disease	N/A	[26], [37]
Qutubuddin et al., 2013	Parkinson's disease and forced exercise: a preliminary study	N/A	[23], [26], [37]
Ribas et al., 2017	Effectiveness of exergaming in improving functional balance, fatigue and quality of life in Parkinson's disease: A pilot randomized controlled trial	NCT02023034	[26], [31], [37]
Schilling et al., 2010	Effects of moderate-volume, high-load lower-body resistance training on strength and function in persons with Parkinson's disease: a pilot study	N/A	[23], [26], [37]
Schenkman et al., 2012	Exercise for people in early- or mid-stage Parkinson disease: a 16-month randomized controlled trial	NCT01257945	[26], [38]
Schenkman et al., 2018	Effect of High-Intensity Treadmill Exercise on Motor Symptoms in Patients With De Novo Parkinson Disease: A Phase 2 Randomized Clinical Trial	NCT01506479	[23], [26]
Shulman et al., 2013	Randomized Clinical Trial of 3 Types of Physical Exercise for Patients With Parkinson Disease	NCT00283504	[23], [26], [37]
Smania et al., 2010	Effect of balance training on postural instability in patients with idiopathic Parkinson's disease: a randomized controlled trial.	N/A	[26], [31]
Tollar et al., 2018	Vastly Different Exercise Programs Similarly Improve Parkinsonian Symptoms: A Randomized Clinical Trial	NCT03193268	[26], [34], [37]
Van der Kolk et al., 2018	A remotely supervised home-based aerobic exercise programme is feasible for patients with Parkinson's disease: results of a small randomised feasibility trial	NTR5434	[23], [42]
Vergara-Diaz et al., 2018	Tai Chi for Reducing Dual-task Gait Variability, a Potential Mediator of Fall Risk in Parkinson's Disease: A Pilot Randomized Controlled Trial	NCT02418780	[34], [37]
Volpe et al., 2014	Comparing the effects of hydrotherapy and land-based therapy on balance in patients with Parkinson's disease: a randomized controlled pilot study	N/A	[26], [37]
Volpe et al., 2017	Water-based vs. non-water-based physiotherapy for rehabilitation of postural deformities in Parkinson's disease: a randomized controlled pilot study	N/A	[26], [37]
Xiao et al., 2016	Effect of health Baduanjin Qigong for mild to moderate Parkinson's disease	N/A	[37], [42]
Yang et al., 2010	Downhill walking training in individuals with Parkinson's disease: a randomized controlled trial	N/A	[26], [31], [37]
Zhang et al., 2015	Effects of Tai Chi and Multimodal Exercise Training on Movement and Balance Function in Mild to Moderate Idiopathic Parkinson Disease	N/A	[26], [34], [37]

**Table 8:** Comparison of AMSTAR 2 reporting adherence between Chinese and non-Chinese research groups

Chinese group study	D1	D2	D3	D4	D5	D6	D7	D8	D9	D10	D11	D12	D13	D14	D15	D16
Zhou 2022 [23]	Y	PY	N	PY	Y	Y	PY	Y	Y	N	PY	N	N	N	Y	Y
Yang 2022 [10]	Y	PY	N	PY	Y	Y	PY	Y	PY	N	PY	Y	N	N	Y	Y
Wu 2021 [25]	Y	PY	N	PY	N	Y	PY	Y	Y	N	Y	N	N	N	Y	Y
Hao 2022 [27]	Y	N	N	PY	Y	N	PY	Y	Y	N	PY	N	N	N	Y	Y
Lei 2022 [28]	Y	PY	Y	PY	Y	Y	PY	Y	Y	N	N	N	Y	N	Y	Y
Tang et al. 2019 [29]	Y	N	N	PY	N	Y	N	Y	Y	N	N	N	N	N	N	Y
Kwok 2022 [30]	Y	Y	Y	Y	Y	Y	PY	Y	Y	N	Y	N	Y	Y	Y	Y
Li 2021 [31]	Y	Y	Y	PY	Y	N	PY	Y	Y	N	N	N	Y	N	N	N
Qian 2023 [33]	Y	Y	Y	PY	Y	Y	N	Y	PY	N	Y	Y	Y	Y	Y	Y
Zhang 2023 [37]	Y	Y	Y	PY	Y	Y	N	Y	PY	N	Y	N	Y	Y	N	Y
Wang 2025 [38]	Y	Y	Y	PY	Y	Y	N	Y	Y	N	Y	N	Y	Y	N	Y
Dou 2025 [39]	Y	Y	Y	PY	Y	Y	N	Y	Y	N	Y	Y	Y	Y	Y	Y
Xie 2025 [40]	Y	Y	Y	PY	Y	Y	N	Y	Y	N	Y	N	Y	Y	N	Y
Fan 2025 [41]	Y	Y	Y	PY	Y	Y	N	Y	Y	N	Y	Y	Y	Y	N	Y
Gao 2024 [42]	Y	PY	N	PY	Y	Y	PY	Y	Y	N	PY	Y	N	PY	PY	Y
Non-Chinese group study																
Chuang 2022 [22]	Y	PY	N	PY	N	N	PY	Y	Y	Y	PY	N	N	N	Y	Y
Álvarez-Bueno 2021 [24]	Y	Y	N	PY	Y	Y	PY	Y	Y	N	Y	N	N	N	Y	Y
Ernst 2023 [26]	Y	PY	N	Y	N	Y	Y	Y	Y	Y	Y	Y	Y	N	Y	Y
Mustafaoglu 2022 [32]	Y	PY	Y	PY	Y	Y	PY	PY	Y	N	Y	N	Y	Y	Y	Y
Lorenzo-García 2024 [34]	Y	Y	Y	PY	Y	Y	N	Y	Y	N	Y	Y	Y	Y	PY	Y
Yau 2024 [35]	Y	Y	Y	PY	Y	Y	N	Y	Y	N	Y	N	Y	Y	Y	Y
Lorenzo-García 2023 [36]	Y	Y	Y	PY	Y	Y	N	Y	Y	N	Y	N	Y	Y	Y	Y
Chinese group Yes	100	53.3	60	6.7	86.7	86.7	0	100	80	0	53.3	33.3	60	46.7	53.3	93.3
Chinese group Partial Yes	0	33.3	0	93.3	0	0	53.3	0	20	0	26.6	0	0	6.7	6.7	0
Chinese group No	0	13.3	40	0	13.3	13.3	46.7	0	0	100	20	66.6	40	46.7	40	6.7
Non-Chinese group Yes	100	57	57	14	71	86	14	86	100	14	86	29	71	57	86	100
Non-Chinese group Partial Yes	0	43	0	86	0	0	43	14	0	0	14	0	0	0	14	0
Non-Chinese group No	0	0	43	0	29	14	43	0	0	86	0	71	29	43	0	0

**Table 9:** Detailed AMSTAR 2 assessment of the 22 included NMAs Critical domains are highlighted in bold. n represents the number of NMAs (N=22) NMAs: Network meta-analyses

AMSTAR 2 Item	Domain Description	Yes n (%)	Partial Yes n (%)	No n (%)
Item 1	PICO components	22 (100.0%)	0 (0.0%)	0 (0.0%)
Item 2 (Critical)	Protocol registration	12 (54.5%)	8 (36.4%)	2 (9.1%)
Item 3	Study design selection	12 (54.5%)	0 (0.0%)	10 (45.5%)
Item 4 (Critical)	Literature search strategy	2 (9.1%)	20 (90.9%)	0 (0.0%)
Item 5	Study selection (duplication)	18 (81.8%)	0 (0.0%)	4 (18.2%)
Item 6	Data extraction (duplication)	19 (86.4%)	0 (0.0%)	3 (13.6%)
Item 7 (Critical)	List of excluded studies	1 (4.5%)	10 (45.5%)	11 (50.0%)
Item 8	Description of included studies	22 (100.0%)	0 (0.0%)	0 (0.0%)
Item 9 (Critical)	Risk of bias assessment	19 (86.4%)	3 (13.6%)	0 (0.0%)
Item 10	Funding sources	2 (9.1%)	0 (0.0%)	20 (90.9%)
Item 11 (Critical)	Statistical methods	13 (59.1%)	5 (22.7%)	4 (18.2%)
Item 12	Risk of bias impact	7 (31.8%)	0 (0.0%)	15 (68.2%)
Item 13 (Critical)	RoB in interpretation	14 (63.6%)	0 (0.0%)	8 (36.4%)
Item 14	Heterogeneity discussion	10 (45.5%)	1 (4.5%)	11 (50.0%)
Item 15 (Critical)	Publication bias investigation	13 (59.1%)	2 (9.1%)	7 (31.8%)
Item 16	Conflict of interest	20 (90.9%)	0 (0.0%)	2 (9.1%)
